# The need for autopsy in all young people dying suddenly including those with epilepsy

**DOI:** 10.1093/europace/euae163

**Published:** 2024-06-13

**Authors:** Nathan A Shlobin, Josemir W Sander, Roland D Thijs

**Affiliations:** Department of Neurosurgery, Neurological Institute of New York, New York Presbyterian Hospital—Columbia University Irving Medical Center, New York, NY, USA; Stichting Epilepsie Instellingen Nederland (SEIN), P.O. Box 540, 2130 AM Heemstede, Netherlands; UCL Queen Square Institute of Neurology, University College London, London WC1N 3BG, UK; Chalfont Centre for Epilepsy, Chalfont St Peter, London, UK; Stichting Epilepsie Instellingen Nederland (SEIN), P.O. Box 540, 2130 AM Heemstede, Netherlands; UCL Queen Square Institute of Neurology, University College London, London WC1N 3BG, UK; Chalfont Centre for Epilepsy, Chalfont St Peter, London, UK; Department of Neurology, West China Hospital, Sichuan University, Chengdu 610041, China; Stichting Epilepsie Instellingen Nederland (SEIN), P.O. Box 540, 2130 AM Heemstede, Netherlands; UCL Queen Square Institute of Neurology, University College London, London WC1N 3BG, UK; Chalfont Centre for Epilepsy, Chalfont St Peter, London, UK; Department of Neurology, Leiden University Medical Centre, Albinusdreef 2, 2333 ZA Leiden, Netherlands

We read with interest the article by Lynge *et al*. on the need for an autopsy in young people who die suddenly.^1^ We commend the authors for drawing attention to such tragic deaths of young people and their devastating consequences. We aim to add nuance to the categorization of sudden death with attention to sudden unexpected death in people with epilepsy (SUDEP) and provide recommendations for the autopsy of young people with sudden death and subsequent management of family members.

Sudden death may involve cardiac, neurologic, or multifactorial aetiologies. The present document emphasized SCD and sudden arrhythmic death syndrome (SADS), but we were surprised as SUDEP was not mentioned. SUDEP, defined as sudden death not due to identifiable causes in a seemingly healthy individual with epilepsy,^2^ is the most common category of premature death in people with chronic epilepsy.^3^ SUDEP is stratified into definite, probable, possible, and near SUDEP, depending on clinical criteria, presence of an autopsy, and evidence for a competing cause of death, then further subdivided into ‘Plus’ if comorbidities other than epilepsy may have contributed to the death.^2^

As awareness of the phenomenon of sudden death has increased, differentiating SUDEP, SCD, and SADS has become essential for risk stratification of close relatives of people who have died suddenly. SCD, SADS, and SUDEP are heterogeneous states that overlap in risk factors, candidate genes, and pathophysiology. SUDEP primary involves non-cardiac mechanisms of death, mostly convulsive seizure-induced post-ictal apnea-asystole.^4^ In contrast, SUDEP Plus involves a preexisting condition (e.g. coronary insufficiency or genetic mutation predisposing to an arrhythmia) that could have contributed to the death while SCD involves an acute cardiac event. SADS may represent the overlap of SUDEP and SCD given the presence of a documented arrhythmia without clinical findings that explain death. Genes implicated in these states, primarily channelopathies, overlap.^5^ Challenges include a lack of data on SCD and SADS in people with epilepsy, labelling of SCD and SADS as probable SUDEP, and the inability to validly diagnose epilepsy and thus SUDEP after death. Classification of sudden death as SCD/probable SUDEP or SADS/definite SUDEP depends on prior medical records, family history, presence of autopsy, and the specialist making the designation.

The authors advocate for the need for an autopsy in all people under the age of 50 years who die suddenly to identify at-risk relatives. We fully agree with this plea. Per clinical judgment, the postmortem examination should include genetic testing focusing on pathological mutations associated with SUDEP, SCD, SADS,^6,7^ and other potentially relevant genes. High-risk relatives should undergo a complete clinical assessment and comprehensive cardiac testing.^7^ Genetic testing should be considered if postmortem genetic testing in the decedent detected a likely pathogenic or pathogenic variant.^8^ We appreciate that the authors underscored that a multidisciplinary approach is essential while emphasizing the importance of including a neurologist.

We encourage the conceptualization of sudden death as SCD, SADS, and SUDEP to improve the diagnostic process for young people with sudden death and facilitate the management of high-risk relatives.
